# Extreme Mountain Ultra-Marathon Leads to Acute but Transient Increase in Cerebral Water Diffusivity and Plasma Biomarkers Levels Changes

**DOI:** 10.3389/fphys.2016.00664

**Published:** 2017-01-05

**Authors:** Davide Zanchi, Magalie Viallon, Caroline Le Goff, Grégoire P. Millet, Guido Giardini, Pierre Croisille, Sven Haller

**Affiliations:** ^1^Department of Psychiatry, University Hospital of BaselBasel, Switzerland; ^2^CREATIS, Centre National de la Recherche Scientifique UMR 5220, INSERM U1206, Université de Lyon, INSA Lyon, Université Jean Monnet Saint-EtienneSaint Etienne, France; ^3^Radiology Department, CHU de Saint EtienneSaint Etienne, France; ^4^Department of Clinical Chemistry, University of LiègeLiège, Belgium; ^5^Institute of Sports Sciences, University of LausanneLausanne, Switzerland; ^6^Department of Neurology and Stroke Unit, Mountain Medicine and Neurology Center Valle d'Aosta Regional HospitalAosta, Italy; ^7^Affidea Centre de Diagnostic Radiologique de Carouge CDRCGeneva, Switzerland; ^8^Faculty of Medicine, University of GenevaGeneva, Switzerland; ^9^Department of Surgical Sciences, Radiology, Uppsala UniversityUppsala, Sweden; ^10^Department of Neuroradiology, University Hospital FreiburgGermany

**Keywords:** ultra-marathon, extreme sport, MRI, apparent diffusion coefficient, brain

## Abstract

**Background:** Pioneer studies demonstrate the impact of extreme sport load on the human brain, leading to threatening conditions for athlete's health such as cerebral edema. The investigation of brain water diffusivity, allowing the measurement of the intercellular water and the assessment of cerebral edema, can give a great contribution to the investigation of the effects of extreme sports on the brain. We therefore assessed the effect of supra-physiological effort (extreme distance and elevation changes) in mountain ultra-marathons (MUMs) athletes combining for the first time brain magnetic resonance imaging (MRI) and blood parameters.

**Methods:**This longitudinal study included 19 volunteers (44.2 ± 9.5 years) finishing a MUM (330 km, elevation + 24000 m). Quantitative measurements of brain diffusion-weighted images (DWI) were performed at 3 time-points: Before the race, upon arrival and after 48 h. Multiple blood biomarkers were simultaneously investigated. Data analyses included brain apparent diffusion coefficient (ADC) and physiological data comparisons between three time-points.

**Results:**The whole brain ADC significantly increased from baseline to arrival (*p* = 0.005) and then significantly decreased at recovery (*p* = 0.005) to lower values than at baseline (*p* = 0.005). While sodium, potassium, calcium, and chloride as well as hematocrit (HCT) changed over time, the serum osmolality remained constant. Significant correlations were found between whole brain ADC changes and osmolality (*p* = 0.01), cholesterol (*p* = 0.009), c-reactive protein (*p* = 0.04), sodium (*p* = 0.01), and chloride (*p* = 0.002) plasma level variations.

**Conclusions:**These results suggest the relative increase of the inter-cellular volume upon arrival, and subsequently its reduction to lower values than at baseline, indicating that even after 48 h the brain has not fully recovered to its equilibrium state. Even though serum electrolytes may only indirectly indicate modifications at the brain level due to the blood brain barrier, the results concerning osmolality suggest that body water might directly influence the change in cerebral ADC. These findings establish therefore a direct link between general brain inter-cellular water content and physiological biomarkers modifications produced by extreme sport.

## Introduction

Mountain ultra-marathons (MUMs) have become increasingly popular in the last decade and they are considered a model to investigate acute responses to extreme load and stress (Millet and Millet, 2012). As previous studies demonstrate, during and immediately after running, MUMs induce cardiac (Maufrais et al., 2016) and neuromuscular (Saugy et al., 2013) fatigue, lung functions alterations as pulmonary ventilations (Vernillo et al., 2015) as well as impairment in postural control (Degache et al., 2014). In parallel, an increase in total body water (Knechtle et al., 2008), in volumes of athletes' body extremities (e.g., feet) (Bracher et al., 2012) and the development of peripheral oedema have been identified (Zavorsky et al., 2014). Blood biomarkers changes also confirm this response, showing higher C-reactive protein concentrations (Millet et al., 2011a; Saugy et al., 2013) and decreased hematocrit levels in runners right after MUMs (Robach et al., 2014; Vitiello et al., 2015).

Besides these physiological changes, pioneer studies demonstrate the impact of extreme sport load also on the human brain. Disorders of serum-sodium occur commonly in athletes participating in endurance sports leading to threatening conditions for athletes health as cerebral edema and pulmonary edema (Ayus et al., 2000). The investigation of brain water diffusivity, allowing the measurement of the intercellular water and the assessment of cerebral edema, can therefore give a great contribution to the investigation of the effects of extreme sports on the brain (Hagen et al., 2007). Rapid diagnosis and appropriate therapy of these athletes is required to prevent severe complications or death.

In the present study, we investigated changes occurring during and after ultra-marathon in brain water diffusivity aiming at investigating the presence of cerebral edemas. In particular, by experience and observation, 48 h is the period post- MUM where the edemas (especially in the calves) are maximal.

Using brain magnetic resonance imaging (MRI) apparent diffusion coefficient (ADC) combined with physiological parameters, the present work aims to (1) investigate the effects of MUM on brain water diffusivity and (2) further explore non-invasively the interaction between MUM-induced body and brain water responses on master runners. This allows us to capture the complete picture of brain and physiological changes generated by endurance running.

## Materials and methods

We performed a serial investigation of MUM athletes at baseline, upon arrival and after 48 h including brain MRI and multiple blood parameters. Brain MRI apparent diffusion coefficient (ADC) derived from diffusion weighed imaging (DWI) was used, which measures the diffusion of water notably in the inter-cellular compartment. As we assume that there will be no significant neuronal axonal growing or shrinking within the short observational interval of less than 1 week, ADC provides an indirect *in-vivo* estimation of the inter-cellular compartment of the human brain. Additionally, we assessed multiple blood parameter covering inflammation, osmolality and electrolytes, and correlated brain ADC with these physiological parameters.

### Subjects and experimental study design

This study was approved by the local ethical committee (Aosta Valley, Azienda USL 101/946), and the experiments were conducted in accordance with the Helsinki Declaration (2001). Subjects were recruited through mailing and public announcements to registered runners by race organizers. Common exclusion criteria were used including smoking, substance abuse, regular intake of medications, medical or psychiatric illness, and any contraindication to Magnetic resonance imaging (MRI) (e.g., claustrophobia, non-removable metal devices) or abnormalities detected upon laboratory screening. Of the 51 experienced runners that volunteered and provided informed written consent to participate in this study, the final sample included 19 healthy male volunteers (44.2 ± 9.5 years) that completed the race with brain-MRI and biological data at all time points. Demographics are shown in Table 1.

**Table 1:** **Demographic and training profile data**.

	**Mean ± *SD***	**Pre**	**Arrival**	**Recovery**	**(P)**
Training/week (Km/week)	44 ± 19.2				
Running experience (years)	12.16 ± 8.2				
Experience in ultramarathons (years)	5.8 ± 3.9				
Previous ultramarathons (*n*)	14.3 ± 16.1				
Height (cm)		176.3 ± 6.2	176.1 ± 6.2	176.1 ± 6.2	/
Weight (kg)		72.6 ± 7.5	72.3 ± 7.4	71.7 ± 8	/

*Demographics. All values are provided as mean (standard deviation). Pre, Arrival, and Recovery was the three key timepoints measurement sessions. No significant differences were found in height and weight for the three timepoints*.

The Tor des Geants® is a 330 km long ultra-distance trail running, with considerable positive/negative elevation changes (+24,000 m) in the Valley of Aosta (Italy). It is considered as one if not the most difficult mountain marathon race in the world since the ultra-endurance activity is associated to high altitude exposure and sleep deprivation. The altitude along the course ranges between 322 and 3,300 m, with 25 mountain passes over 2000 m. The maximum time allowed to complete the race is 150 h, and in 2014, the best performance was 71 h 49 min among 740 starters and 446 (60%) finishers (http://www.tordesgeants.it/). Before the race weight and height of the subjects were measured. Moreover, the participants were interviewed about the training weeks (km/week) the running experience (years), the experience in ultra-marathons (years) and the number of previous marathons run.

The present experimental design was a longitudinal study with repeated assessments at three key time points. The first session (pre-race: T1) was performed within 4 days before the race; the second at the end of the race (arrival: T2): athletes were brought by car to the laboratory and were evaluated in less than 1 h after the arrival; a last session (recovery: T3) was finally performed after 48–72 h after arrival time. Blood analyses, body temperature (°C) and Diffusion-Weighted Imaging (DWI) examination were recorded at each session. Blood examination included assessment of hematocrit (HCT) (%), lactate (mmol/L), C-reactive protein (CRP) (mg/L), urinary creatinine (g/L), creatinine (mg/dL), calcium (mmol/L), chloride (mmol/L), potassium (mmol/L), sodium (mmol/L), phosphates (mmol/L), osmolality (mosm/kg), urinary osmolality (mosm/kg), cholesterol (mg/dL).

### DWI acquisition

Images were obtained using a 1.5T scanner (MAGNETOM Avanto, Siemens Healthcare, Erlangen, Germany) with a standard 12 channel head-coil. DWI imaging of the whole brain was acquired using standard parameters (Viallon et al., 2015) whole brain coverage (34 slices, slice thickness 4 mm), parallel imaging (grappa factor = 2), 2 values (b = 0.1000). Main MR parameters were: TE = 95 ms, TR = 5500 ms, 4 averages, none interpolated pixel size 1.4375 × 1.4375, acquisition matrix 160 × 160, FOV 230 mm.

### Laboratory analyses

Blood samples were collected at each session within 10 min after arrival at each key point. Samples were drawn from an antecubital vein into a dried, heparinized or EDTA tube according to the analysis to be performed. Both tubes were immediately centrifuged for 10 min (3500 RPM). Since it was not possible to carry out all the analyses on the same day by point-of care technologies, plasma and serum were frozen at −80°C within 20 min after blood collection for later analysis of muscle injury markers and biochemical variables. The hematology parameters (hemoglobin, red blood cell, white blood cell) were directly analyzed by pocH-100i™automated hematology analyzer (Sysmex, Villepinte, France). Cobas 8000 (RocheDiagnostics, Manheim, Germany) were used to perform serial determinations for C-reactive protein (CRP), urinary creatinine, creatinine, calcium, chloride, potassium, sodium, and cholesterol. The osmolality and urinary osmolality were measured on ARKRAY OSMO STATION OM-6050 (Menarini, Florence, Italy).

### Statistical analysis

The statistical analyses were conducted using GraphPad Prism (Version 6, GraphPad Software, San Diego, USA) and FSL (Version 5.0.9, FMRIB, Oxford, UK). Subjects' height and weight values were submitted to a repeated measure ANOVA using Tuckey correction for multiple comparisons.

### Group analyses of DWI data

FSL software was used to analyze diffusion weighted imaging (DWI) data. First, brain extraction and tissue-type segmentation were conducted using the corresponding FSL tools (Brain Extraction Tool and FAST4) (Smith, 2002; Smith et al., 2004). Then, a non-linear transformation into Montreal Neurological Institute (MNI) reference space was applied and a study-specific ADC template was created. The images were smoothed with an isotropic Gaussian kernel of 5 mm sigma. Finally, to investigate differences in ADC between T1, T2, and T3, a permutation-based non-parametric test (randomize, FSL tool) was applied, correcting for multiple comparisons by threshold-free cluster enhancement (Winkler et al., 2014). *P*-values < 0.05 were considered as significant.

### *Post-hoc* analyses of DWI data

After ADC data preprocessing, randomize was run to test for differences in handiness and footedness in the subjects at T1, T2, and T3 separately in order to exclude potential confounding effects by left/right-handed participants at the brain level. Furthermore, differences in specific brain regions were also tested at the three time-points. After preprocessing, masks of frontal, parietal, and occipital areas were applied to the subjects' normalized individual brain and respective ADC values were extracted. Finally repeated measures single-factor analysis of variance (ANOVA) was performed using Geisser-Greenhouse estimates to account for sphericity violation. *Post-hoc* multiple comparisons were performed using Tukey's corrections.

### Analysis of biological data

To assess the effect of ultra-endurance exercise on biological data at the different times (baseline, arrival, recovery) pairwise repeated measures single-factor ANOVA models were performed for each variable using Geisser-Greenhouse correction for sphericity violation and Tukey *post-hoc* pair-wise multiple comparisons corrections.

### Correlation blood biomarkers–ADC values

First, averaged whole brain ADC signal for each subject was extracted separately. Then, Pearson correlations were computed between the delta of ADC values and of the blood biomarkers concentrations (T2–T1 and T3–T2). False discovery rate (FDR) was used to correct for multiple comparisons.

## Results

### Demographics

The mean time to finish at T2 was 86 h, 49 min 20 s, standard deviation = 45 h, 17 min, 15 s. The subjects were “Master runners,” running an average of 44 ± 19.2 km/week, having 12.2 ± 8.2 years of running experience and having already run a mean of 14.3 ± 16.1 MUMs for an average of 5.8 ± 3.9 years. Loss in weight and height from T1 to T3 were shown even though the difference after ANOVA is not statistically significant (Table 1).

### DWI results

**Figure 1:**
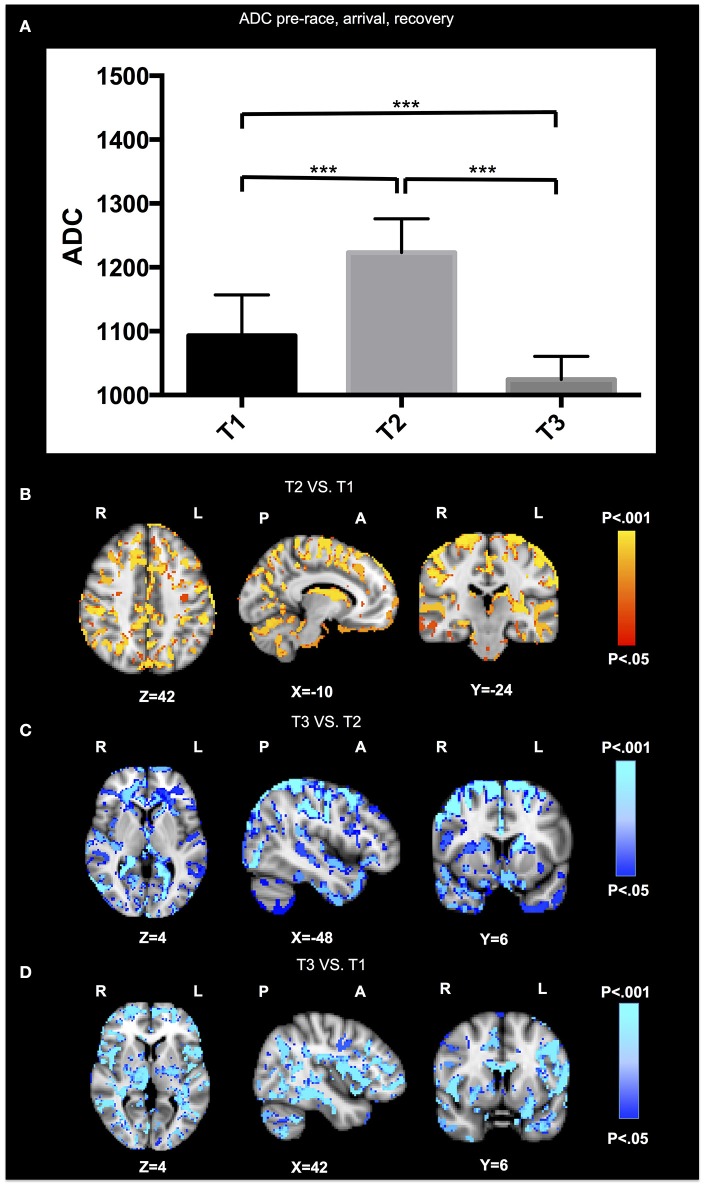
**DWI results**. The average ADC value across the entire brain **(A)** increased from T1 to T2 (*p* = 0.005), and then decreased from T2 to T3 (*p* = 0.005), while the values at T3 were actually below the baseline T1 (*p* = 0.005). The voxel-wise analysis are illustrated for the contrasts of T2 vs. T1 **(B)**, T3 vs. T2 **(C)**, and T3 vs. T1 **(D)**. No significant differences in ADC signal were found for the inverse comparisons. Radiologic convention, right hemisphere on left hand side. Results of ADC analysis are super-imposed on the Montreal Neurologic Institute (MNI) standard brain.

The average ADC value across the entire brain increased from T1 (1093 ± 64.1) to T2 (1223 ± 52.9) (*p* = 0.005), and then decreased from T2 to T3 (1024 ± 36.4) (*p* = 0.005). The values at T3 were actually below the baseline T1 (*p* = 0.005). The voxel-wise analysis confirmed these results, and revealed no significant voxels for the inverse comparisons (Figure 1).

### *Post-hoc* analyses of DWI data

No significant differences were found in subjects for handiness and footedness at the three time-points. Moreover, no significant differences between ADC values in different brain areas were found for the three time-points.

### Biological results

Significant higher concentrations at baseline (T1) compared to arrival (T2) and recovery (T3) were found for HCT (*F* = 93.9, *p* < 0.001), cholesterol (*F* = 123.4, *p* < 0.001), and calcium (*F* = 82.2, *p* < 0.001). A significant increase in concentration at arrival (T2) compared to baseline (T1) and recovery (T3) was found for CRP (*F* = 20.4, *p* < 0.001) and urinary osmolality (*F* = 9.1, *p* < 0.01). A significant increase in concentration at recovery (T3) compared to baseline (T1) and arrival (T2) was found for chloride (*F* = 11.1, *p* < 0.001).

Significant differences were found in creatinine between baseline (T1) and recovery (T3) and arrival (T2) and recovery (T3) (*F* = 11.2, *p* < 0.01). Significant differences were found in potassium (*F* = 18.2, *p* < 0.001) between baseline (T1) and arrival (T2) and arrival (T2) and recovery (T3) and in phosphates (*F* = 9.7, *p* < 0.01).

No significant differences between T1, T2, and T3 were found for the other physiological variables.

**Figure 2:**
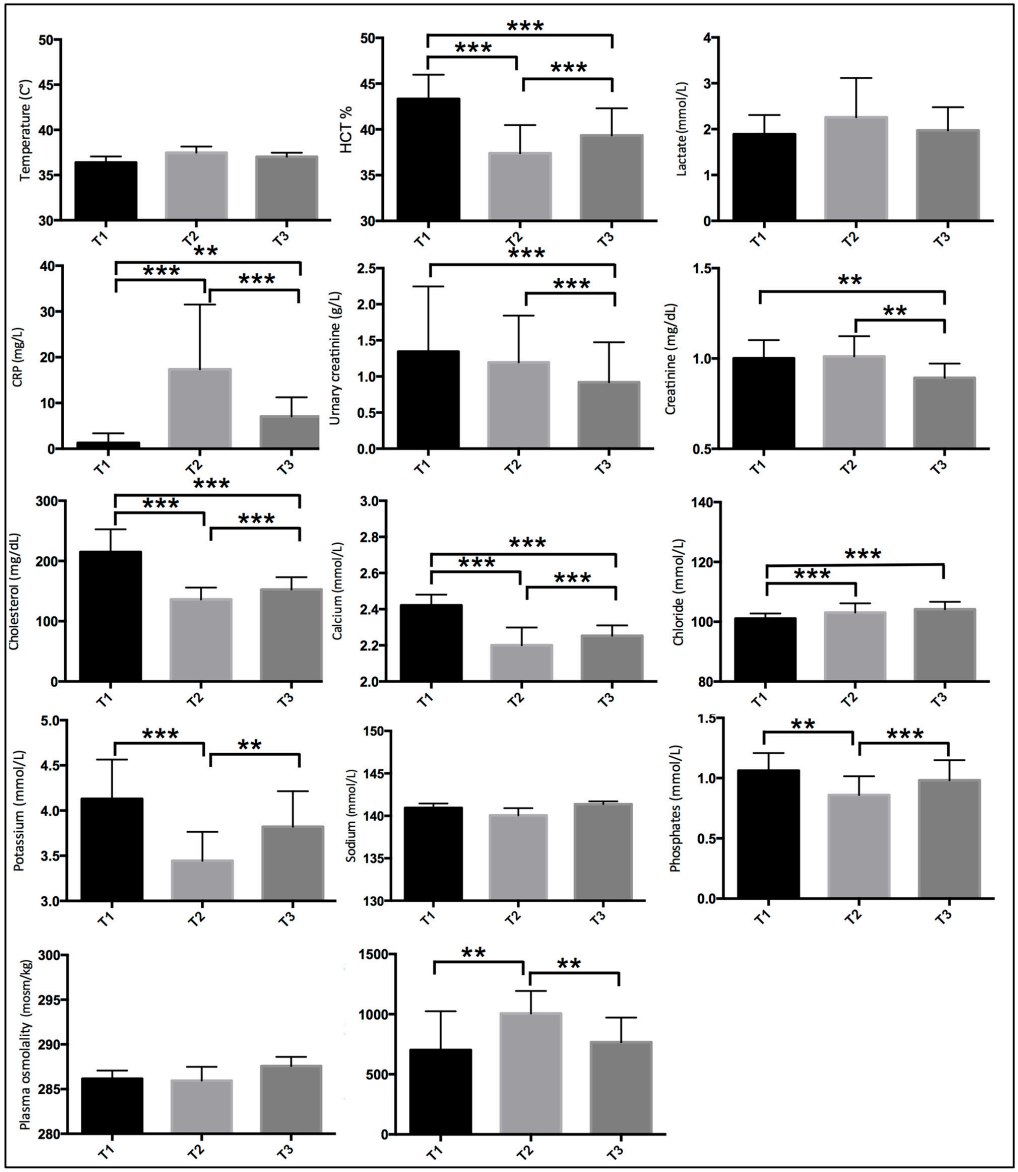
**Plasma biomarker concentrations during pre-race (T1), arrival (T2), and recovery (T3)**. ^*^ indicates *p* < 0.05, ^**^ *p* < 0.01, ^***^p < 0.001.

Differences in plasma biomarker concentrations at the three time-points are shown in Figure 2.

### Correlation blood biomarkers–ADC values

**Figure 3:**
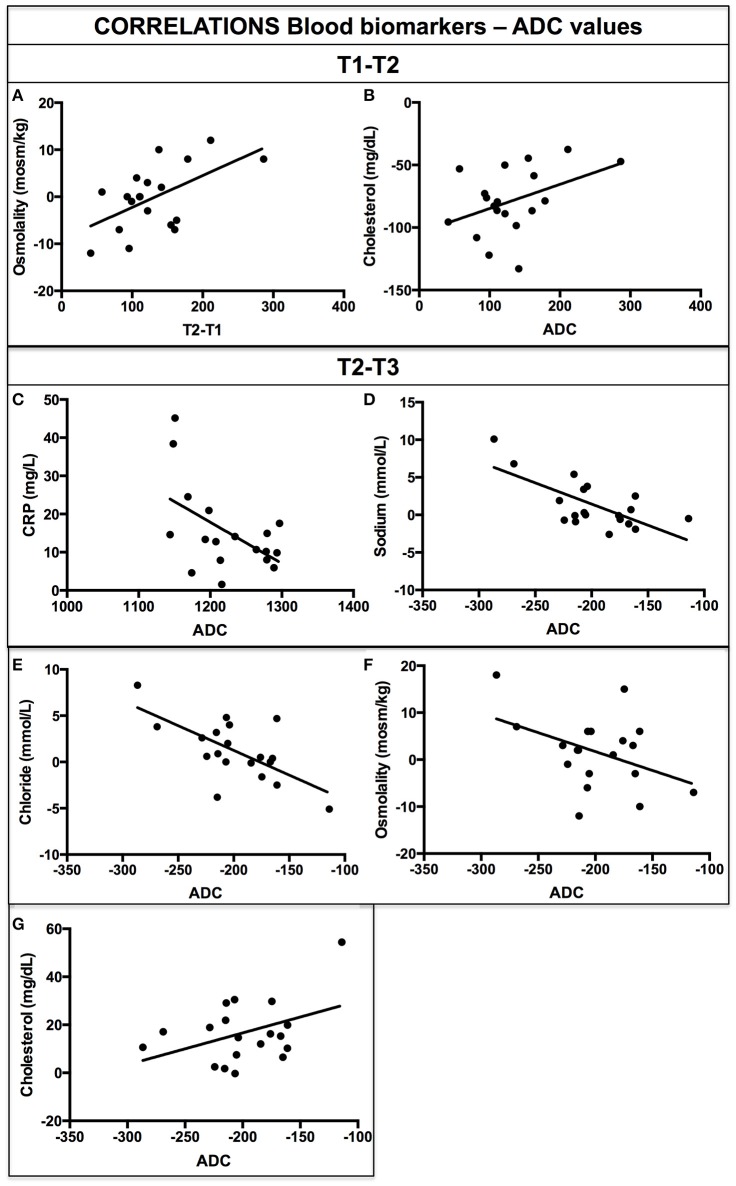
**Correlations delta blood biomarkers and ADC values for T2–T1 (A,B)** and for T2–T3 **(C–G)**.

No correlations survived FDR multiple comparison correction. Non-corrected significant positive correlations were found between changes in brain ADC values and in osmolality (*r* = 0.56, *p* = 0.01) plasma levels from T1 to T2 (Figure 3A). A correlation that approaches the significant level (*r* = 0.41; *p* = 0.07) as found between AD and cholesterol plasma levels (Figure 3B).

Moreover, significant negative correlations were found between changes from T1 to T2 in brain ADC values and in CRP (*r* = −0.58; *p* = 0.009) (Figure 3C), sodium (*r* = −0.69; *p* = 0.001) (Figure 3D), chloride (*r* = −0.66; *p* = 0.002) (Figure 3E) plasma levels and in osmolality (*r* = −0.42; *p* = 0.07) and urinary osmolality approaching the significance level (*r* = −0.41; *p* = 0.07) (Figure 3F). A positive correlation was found between changes in ADC values and in Cholesterol (*r* = 0.41; *p* = 0.04) from T1 to T2 (Figure 3G). No other significant correlations were found between ADC values and other blood biomarkers concentration.

## Discussion

The current investigation assessed the effect of extreme effort (both extreme distance and elevation changes) in mountain ultra-marathons (MUMs) athletes on brain water diffusion and blood physiological parameters in master runners. The whole brain ADC significantly increased from baseline to arrival and then significantly decreased at recovery, to lower values than at baseline. Linked changes in physiological electrolytes concentration are also present during and after MUM. Our findings identify the presence of a brain-body matrix suggesting that together with changes at the body level, modifications at the brain level can be also demonstrated upon arrival, and that even 48–72 h after arrival, the brain has not yet completely recovered to the baseline state.

ADC measures the diffusion of water notably in the inter-cellular space (Duong et al., 2001; Sehy et al., 2002). Two are the main origins for an increase in ADC values: The first is the neuronal and axonal degeneration (Simon and Kliot, 2014) that consists in progressive structural atrophy occurring over years, for example during neurodegenerative diseases (Erickson et al., 2013). This scenario is unlikely in the current setting, since the ADC changes appear in a range of few days.

The second option consists in rapid changes in the inter-cellular space. The typical example is acute stroke (Albers, 1998; van Everdingen et al., 1998) when neurons swell in the acute phase, inter-cellular space is therefore reduced and consequently ADC values decrease (Marks et al., 1996; Birenbaum et al., 2011). In the context of MUM, changes in water and electrolyte concentration may rapidly modify the neural inter-cellular space (Sotak, 2004). Due to the speed of these alterations, this is the most plausible scenario and can be considered parallel to the one occurring at the physical level already reported by previous studies on MUMs.

In fact, after ultra-marathon an increase in total body water (Mattson, 2014), in volumes of athletes' body extremities (e.g., feet) (Bracher et al., 2012) and the development of peripheral oedema have been identified. Following this line of research, our results suggest for the first time the occurrence of an overall increase in inter-cellular water also in the brain of runners immediately after ultra-marathon. Few days after MUM, when the athletes recover, extra-cellular space increases in the whole brain from baseline to arrival and then it decreases overshooting the baseline and not yet completely recovering to the baseline equilibrium state after 48 h.

To further explore this brain-body interaction after extreme sport, we assessed multiple parameters in the peripheral blood. First, we observed a significant increase in CRP (c-reactive protein) upon arrival and a partial recovery after 48–72 h. The increase in inflammation demonstrated after MUMs (Millet et al., 2011a; Saugy et al., 2013) can also explain the increase in total body water because inflammation was shown to be associated with tissue oedema (Proske and Morgan, 2001). Our results are therefore in line with previous works (Kasapis and Thompson, 2005; Stewart et al., 2007) showing that extreme exercise produces a short-term, systemic inflammatory reaction, whereas a long- term “anti-inflammatory” response can be expected. Confirming this interpretation, urine osmolality follows the CRP pattern also increasing and then partially recovering. This reflects an exchange in total body water that can also be explained by dehydration and hydration mechanisms during and after sport (Shirreffs and Maughan, 1998; Oppliger et al., 2005; Popkin et al., 2010).

Moreover, concerning electrolytes, the changes over time can also be compared to the brain ones. In particular, the hematocrit, which measures the percentage of serum vs. corpuscular components of the blood, first decreased and then partially recovered. Coherently with our previous interpretation on ADC results and with previous studies on MUMs the large decrease in HCT from T1 to T2 may reflect a large hemodilution: Expansion in plasma volume and in total extracellular water (Robach et al., 2014; Vitiello et al., 2015).

The pattern of CRP, urinary osmolality and HCT mirrors our previous neuroimaging results reflecting an increase in extra-cellular space from T1 to T2. On the contrary the ADC overshooting beyond the baseline (from T2 to T3) seems to be a peculiar pattern of the brain, not reflected by electrolytes.

On the other side, the electrolytes calcium, chloride, potassium and sodium had different and opposite evolutions over time. While calcium and potassium had a similar pattern decreasing from T1 to T2 and then increasing without reaching the baseline level at T3, chloride presented an opposite pattern, increasing at arrival (T2) and not decreasing anymore. Sodium remained always constant. Our study extents findings of previous studies on MUMs (Millet et al., 2011b; Millet and Millet, 2012; Saugy et al., 2013), showing specific modifications in electrolytes concentration after 48 h of intense sport activity. In particular, an increase in plasma volume due to an increase in the total exchangeable potassium has been already previously reported after endurance events (Milledge et al., 1982; Maughan, 1985; Fellmann et al., 1999; Knechtle et al., 2008).

This complex pattern of the various electrolytes over time (plus the other electrolytes not assessed here) may explain the stability of the serum osmolality.

After measuring brain water diffusion parameters and blood biomarkers, we performed additional correlation analyses to find a direct link between electrolytes variations and brain ADC changes. For T2–T1, an increase in cerebral ADC is associated to higher levels of plasma osmolality and cholesterol. For T3–T2 a decrease in ADC level is negatively associated to CRP, chloride, sodium and osmolality and positively to cholesterol.

We want to highlight that the results concerning osmolality are of great interest confirming our previous interpretation. They suggest that body water might directly influence the change in cerebral ADC, increasing first and then decreasing. They establish therefore a direct link between general brain structural changes and physiological biomarkers modifications produced by extreme sport.

In conclusion, our findings show that MUMs induce in the brain of runners increased inter-cellular water in the brain and increased extra-cellular water in the body.

Finally, we want to highlight the absence of differences for handiness and footedness and after post hoc analyses of ADC. Our interpretation is that during marathon races and extreme sports in general, ADC changes in the brain reflect overall body hemodynamics changes rather than specific changes in brain functions.

## Strengths and limitations

Major strengths of the current investigation include the prospective study design, and the rigorous assessment of both brain MRI and blood samples before, upon arrival and during recovery of extreme mountain marathon athletes. Major limitations include the small sample size and the indirect assessment of brain modifications. Concerning the small sample, this can be a reason why the correlations blood biomarkers-ADC values didn't survive multiple comparisons corrections. Future studies with higher number of participants are recommended. Moreover, the assessed ADC values indirectly assess the diffusion of water dominantly in the inter-cellular compartment of the brain, and serum electrolytes may only indirectly indicated modifications at the brain level due to the blood brain barrier. Furthermore, ADC changes in the brain can be influenced by sleep deprivation during the race, for which we didn't perform any standardized assessment. However, in the absence of more precise and non-invasive assessments at the level of the brain, the observed indirect methods provide at least a feasible approach to assess modifications of the brain in response to extreme physical activity. Concerning the blood sampling, some limitations must be acknowledged. One limitation was the timing of the blood sampling. The samples were not taken at exactly the same time because all the subjects did not run at the same speed, even if the sampling was made at identical moment of the Tor. Moreover, intra-individual biological variations exist which can have an impact on the results. The post-exercise liquid intake could also lead to biological modifications due to a possible hemodilution but as the hematocrit didn't change a lot, we conclude that the impact of this potentially confounding factor is very low. The subjects were allowed to consume food and fluid *ad libitum* during the race, but the intake was not monitored due to logistical constraints and the difficulty to access the runners on mountainous path along the MUM (Maufrais et al., 2016). Furthermore, the exact weather conditions during the race were not monitored. Finally, having diet, exercise, caffeine intake values before even before T1 and after T3 would be a great improvement for our study. Unfortunately, most runners were not locals and came the day of the race and left the day after the study. It was very difficult to control for these factors.

## Conclusion

The current investigation assessed the effect of extreme effort (both extreme distance and elevation changes) in MUMs athletes on brain water diffusion and blood physiological parameters. Extreme physical activity leads to a significant increase of the inter-cellular compartment of the brain, which is not at the equilibrium state even 48–72 h after the race.

## Authors contributions

Conceived and designed the experiments: MV, SH, PC. Performed the experiments: MV, PC. Data analyses: DZ, SH, and MV. Blood analyses: CL. Manuscript writing: DZ, SH, and MV. Manuscript editing: PC, GM, GG, and CL. Ethical Commitee and whole project organization: GG, GM, MV, and PC.

### Conflict of interest statement

The authors declare that the research was conducted in the absence of any commercial or financial relationships that could be construed as a potential conflict of interest.

## Abbreviations

ADC: Apparent diffusion coefficient
ANOVA: Analysis of variance
CRP: C-reactive protein
DWI: Diffusion weighed imaging
FDR: False discovery rate
FSL: FMRIB Software Library
HCT: Hematocrit
MNI: Montreal neurological institute
MRI: Magnetic resonance imaging
MUMs: Mountain ultra-marathons.
